# A Case of Severe Mitral Valve Regurgitation in a Patient with Leadless Pacemaker

**DOI:** 10.1155/2020/5389279

**Published:** 2020-06-30

**Authors:** Srikala R. Gumireddy, Minako Katayama, Hari P. Chaliki

**Affiliations:** Division of Cardiovascular Diseases, Mayo Clinic, Scottsdale, Arizona, USA

## Abstract

An 85-year-old man with cardiac history notable for atrial fibrillation diagnosed 10 years ago which was being treated with atenolol and warfarin presented to our institution with persistent atrial fibrillation. His echocardiogram showed ejection fraction (EF) of 56%, no regional wall motion abnormalities, mild mitral and pulmonary regurgitation, and trivial tricuspid regurgitation. Despite this treatment, he had recurrent episodes of paroxysmal symptomatic atrial fibrillation with a rapid rate requiring multiple emergency department visits and hospital admissions. Given difficulty to control the rate, he underwent atrioventricular (AV) nodal ablation and leadless pacemaker insertion. Fifteen days after the procedure, he was found to have a severe mitral regurgitation murmur.

## 1. Introduction

Leadless pacemakers have been shown to have lesser risk of complications such as pocket infections, endocarditis, pacemaker-induced tricuspid regurgitation, and tamponade. However, this newer technology can lead to significant mitral regurgitation due to iatrogenic LBBB and left ventricular (LV) dyssynchrony.

## 2. Case Presentation

An 85-year-old man presented to our institution complaining of recurrent fever and a mitral regurgitation murmur. He has a past history significant for atrial fibrillation being treated with apixaban status post leadless pacemaker implantation and AV nodal ablation 15 days ago, hypertension, hyperlipidemia, obstructive sleep apnea on continuous positive airway pressure, diverticulosis and diverticulitis, dental caries, melanoma in situ, and persistent pulmonary coccidioidomycosis on fluconazole.

**Figure 1:**
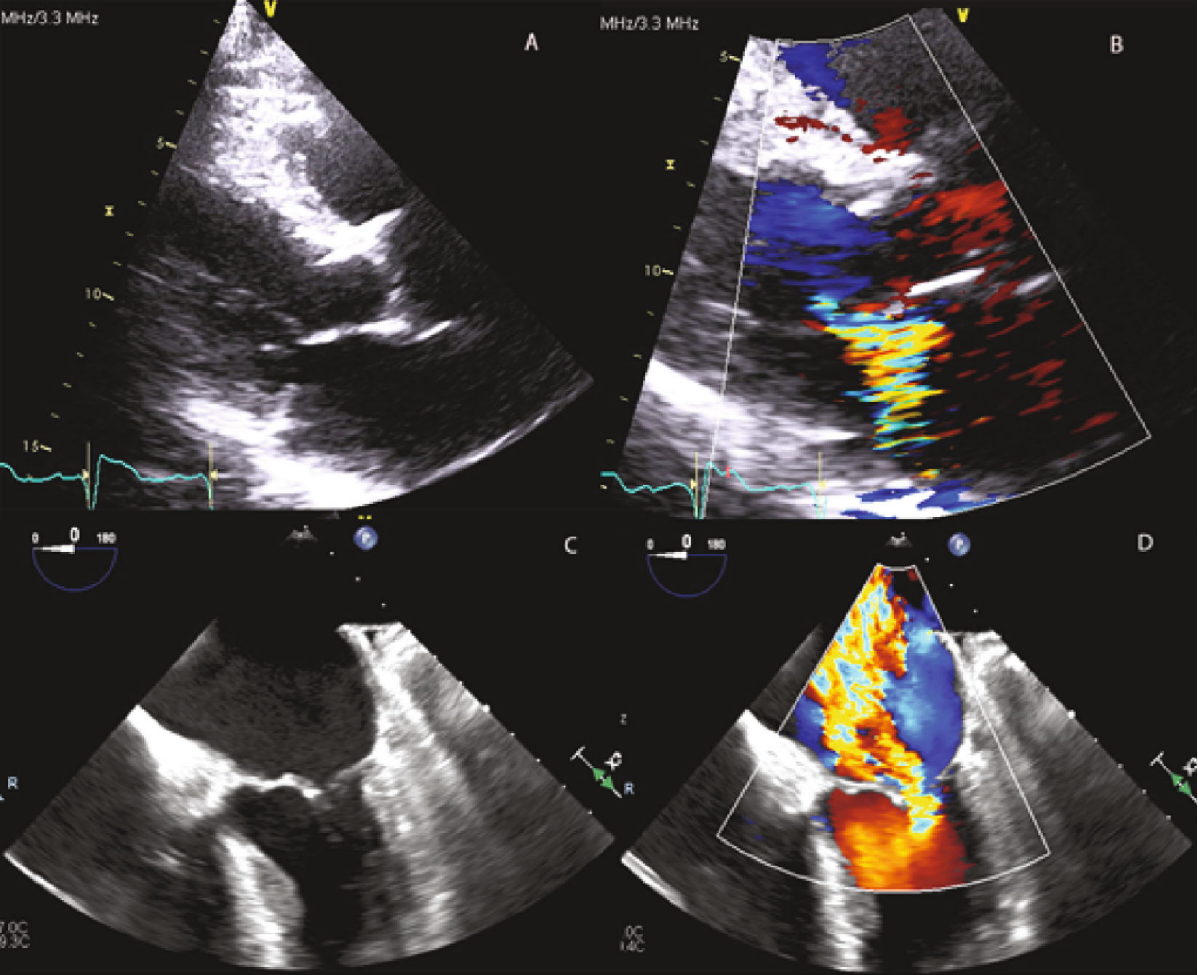
Transthoracic and transesophageal echocardiograms after leadless pacemaker insertion. (a) A transthoracic echocardiogram (parasternal long) taken after insertion of the leadless pacemaker showing incomplete coaptation of mitral valve leaflets. (b) A transthoracic echocardiogram with color (parasternal long) showing severe mitral regurgitation (effective regurgitant orifice area of 0.51 cm^2^ and regurgitant volume of 52 ml) after leadless pacemaker insertion. (c) A transesophageal echocardiogram showing incomplete coaptation of mitral valve leaflets following leadless pacemaker implantation. (d) A transesophageal echocardiogram with color showing severe mitral regurgitation following leadless pacemaker implantation.

He underwent a transthoracic echocardiogram which showed normal left ventricular size and no wall motion abnormalities, LVEF of 56%, severe mitral regurgitation due to incomplete coaptation of the mitral valve leaflets (Figures 1(a) and 1(b)), mild right ventricular enlargement, systolic dysfunction with visible apical right ventricular lead, mild tricuspid regurgitation, and no pericardial effusion. A transesophageal echocardiogram confirmed restricted mitral valve leaflets resulting in incomplete coaptation and severe mitral regurgitation (Figures 1(c) and 1(d)). No vegetations were seen. Blood cultures ruled out endocarditis as the cause of fever, and he was later found to have non-Hodgkin's lymphoma.

**Figure 2:**
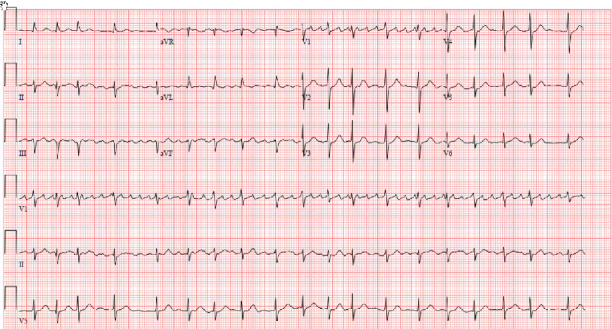
ECG prior to leadless pacemaker insertion. An ECG demonstrating atrial fibrillation with a rapid ventricular rate prior to insertion of the leadless pacemaker.

**Figure 3:**
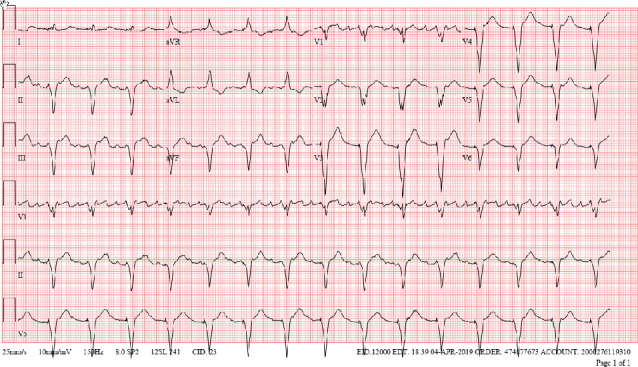
ECG after leadless pacemaker insertion. An ECG taken after insertion of the leadless pacemaker that demonstrates LBBB.

**Figure 4:**
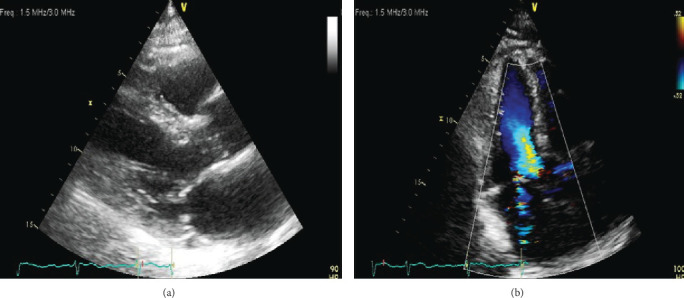
Echocardiograms 25 days prior to leadless pacemaker insertion. (a) A transthoracic echocardiogram (parasternal long) prior to leadless pacemaker insertion showing normal coaptation of mitral valve leaflets. (b) An apical 4-chamber view taken prior to leadless pacemaker insertion showing only mild mitral regurgitation.

His cardiac history is notable for atrial fibrillation diagnosed 10 years ago which was treated with atenolol and warfarin. Despite this treatment, he had recurrent episodes of paroxysmal symptomatic atrial fibrillation with a rapid rate requiring multiple emergency department visits and hospital admissions (Figure 2). Given difficulty to control the rate, he underwent AV nodal ablation and leadless pacemaker insertion 15 days prior to current presentation with resultant LBBB on his electrocardiogram (ECG) post implantation (Figure 3). His echocardiogram days before pacemaker insertion revealed only mild mitral regurgitation and normal coaptation of the mitral valve leaflets (Figure 4).

Given the absence of degenerative mitral valve abnormality and no evidence of cardiomyopathy, his diagnosis of new-onset severe mitral regurgitation is the direct result of abnormal electrical conduction related to iatrogenic LBBB due to AV nodal ablation and single-chamber paced rhythm.

## 3. Discussion

Mitral regurgitation in majority of cases is related to primary mitral valve diseases such as mitral valve prolapse, endocarditis, or rheumatic heart disease which was not the case in our patient [1]. Mitral regurgitation can also occur due to secondary causes related to left ventricular abnormalities such as ischemic or dilated cardiomyopathy which were also not present in our patient [1]. More recently, it has been recognized that long-standing atrial fibrillation can result in atrial cardiomyopathy and annular dilatation leading to mitral regurgitation [2] which was unlikely in our case because he did not have severe mitral regurgitation prior to his pacemaker implantation. In addition, there has been a recent study indicating worsening of mitral regurgitation in patients twelve months following leadless pacemaker implantation [3].

In conclusion, given the prior absence of mitral valve abnormality and cardiomyopathy, this diagnosis of new-onset severe mitral regurgitation is the direct result of abnormal electrical conduction related to iatrogenic LBBB [4] due to AV nodal ablation and single-chamber paced rhythm. Biventricular pacing should be considered when mitral regurgitation occurs post leadless pacemaker insertion.

## Additional Points

*Learning Objective*. This case highlights the need for careful consideration of etiology for mitral regurgitation following leadless pacemaker implantation. Our patient's severe mitral regurgitation is the direct result of abnormal electrical conduction related to iatrogenic left bundle branch block (LBBB) due to AV nodal ablation and single-chamber paced rhythm. It is important to recognize this entity as such patients will benefit from biventricular pacing rather than surgery or mitral clip.
